# Effect of Y_2_O_3_ Addition on Microstructure and Properties of Laser Cladded Al-Si Coatings on AZ91D Magnesium Alloy

**DOI:** 10.3390/ma16010338

**Published:** 2022-12-29

**Authors:** Xiaofeng Wan, Chuang Tian, Yi Li, Jingling Zhou, Shuangqing Qian, Lihong Su, Li Wang

**Affiliations:** 1 School of Mechanical and Manufacturing Engineering, Nantong University, Nantong 226019, China; 2 School of Mechanical, Materials, Mechatronic and Biomedical Engineering, University of Wollongong, Wollongong, NSW 2522, Australia

**Keywords:** AZ91D magnesium alloy, laser cladding, Al-Si + Y_2_O_3_ coating, tribological property, wear resistance, corrosion resistance

## Abstract

The effect of Y_2_O_3_ addition on the microstructure and properties of the laser cladded Al-Si alloy coating on the surface of AZ91D magnesium alloy was investigated in this study. The experimental results showed that the Al-Si + Y_2_O_3_ cladding layers contained α-Mg, Mg_2_Si, Al_4_MgY and a small amount of Al_12_Mg_17_ phases. The coarse dendrites, reticulated eutectic structures and massive phases in the coatings tended to be refined and gradually uniformly distributed with the increased amount of Y_2_O_3_. The introduction of Y_2_O_3_ into the cladding layer favored the improvement of microhardness and wear resistance due to the grain refinement strengthening and dispersion strengthening. The addition of Y_2_O_3_ also promoted the reduction of localized corrosion sites and made the corrosion surface smoother, implying that the corrosion resistance of the Y_2_O_3_-modified coatings was better than that of the unmodified cladding layer.

## 1. Introduction

Magnesium alloy has the advantages of high specific strength and low density, and is currently a common light metal material [1,2,3]. In recent years, it has been widely used in the fields of automobile, aviation, national defense, medical and electronics [4,5,6]. Other benefits of magnesium alloys include their high machinability, good castability, hot formability, and recyclable nature [7,8,9]. However, the surface performance of magnesium alloys is typically poor due to high chemical reactivity, weak hardness, poor corrosion resistance, and poor wear characteristics, which restricts their wide range of applications in various industries [10,11,12,13]. According to Abbas et al. [10], the wear rate of as-received magnesium alloy was greatly higher than the samples subjected to a laser remelting treatment. In the report by Liu and Guo [13], both microstructure and mechanical properties of the magnesium alloy fabricated by selective laser melting were dependent on the heat treatment conditions, such as the temperature and heat treatment duration.

In order to increase the wear and corrosion resistances of magnesium alloy, surface treatments such laser surface cladding [14,15,16], chemical plating [17], electroplating [18], and anodic oxidation are some effective methods [19,20], among which the laser surface cladding technique has garnered a significant amount of interest recently [21,22,23]. For example, a coating with TiC reinforce successfully enhanced the microhardness and wear resistance by about 5–6 and 4.5–5.8 times [16]. By applying Al-Si powders to three distinct magnesium alloys (AZ61, WE54 and ZK30) with a laser of Nd:YAG, Bernabe et al. [2] obtained coatings with no cracks, no pores, and strong metallurgical bonding. Lei et al. [24] produced dense, crack and porous-free Al-Si coatings with a saw-tooth form and strong metallurgical bonding on the surface of AZ91D magnesium alloy. Al + Al_2_O_3_ powders were laser-cladded onto a magnesium alloy surface by Hazra et al. [25], which resulted in decreased wear rate relative to the magnesium alloy substrate.

Laser cladding experiments on magnesium alloy surfaces to improve their properties have a mature technical foundation, and the addition of rare earth oxides plays an invaluable role in the modification of original powders in laser cladding. Zhu et al. [26] studied the impact of the addition of Y_2_O_3_ on the surface of magnesium alloy on Al-Cu cladding coating. Their findings demonstrated that Y_2_O_3_ considerably improved the mechanical characteristics and gave rise to a microstructure that was dendritic, striped, or dispersed granular. Yang and co-authors [27] prepared Al-TiC-Al_3_Ti composites containing Y_2_O_3_ (0–2 wt.%) on AZ91D matrix by laser cladding. When an appropriate amount of Y_2_O_3_ was introduced into the coating, it is found that the TiC phase within the composite coating is fine and dispersed. The addition of Y_2_O_3_ enhances the mechanical properties and corrosion resistance of the cladding. By using laser surface cladding, Bu et al. [28] successfully created Al-TiC-Y_2_O_3_ composite coating on the surface of AZ63-Er alloy. The results showed that the hardness of the cladding layer containing Y_2_O_3_ is higher than that without adding Y_2_O_3_, and has the highest corrosion resistance as it contains 0.6% Y_2_O_3_. At present, there are very few studies on yttrium oxide modification of Al-Si coating. In this study, therefore, the effect of adding different amount of Y_2_O_3_ from 1 to 2 wt.% on the microstructure as well as properties of Al-Si laser cladded layer above AZ91D magnesium alloy surface has been analyzed. The properties of the coatings were systematically evaluated in terms of the microhardness measurement, wear and corrosion testing experiments.

## 2. Materials and Methods

### 2.1. Sample Preparation

The AZ91D magnesium alloy employed as the matrix material for this study has a geometry of 100 × 50 × 15 mm^3^. Table 1 displays the AZ91D chemical composition. The primary coating powder is Al-12Si (wt.%, the same as following). The Al-12Si powder has a size in the range from 100 to 150 μm. The rare earth oxide Y_2_O_3_ (99.99% purity and 75 µm mean powder size) with 1.0, 1.5 and 2.0 wt.%, respectively, has been incorporated into the primary powders to investigate how it affects the coatings microstructure and properties. Four groups of cladding powders, illustrated in Table 2, were created.

Before laser cladding, use 60 to 1500 mesh metallographic sandpaper to grind the surface of the substrate, wash away the abrasive debris with deionized water and dry it. The surface was then roughened by sandblasting with Al_2_O_3_, and finally ultrasonically cleaned with acetone, alcohol and deionized water in sequence. At least three specimens per group were made in order to evaluate performance and study microstructure and to acquire accurate data.

### 2.2. Laser Cladding Process

**Figure 1:**
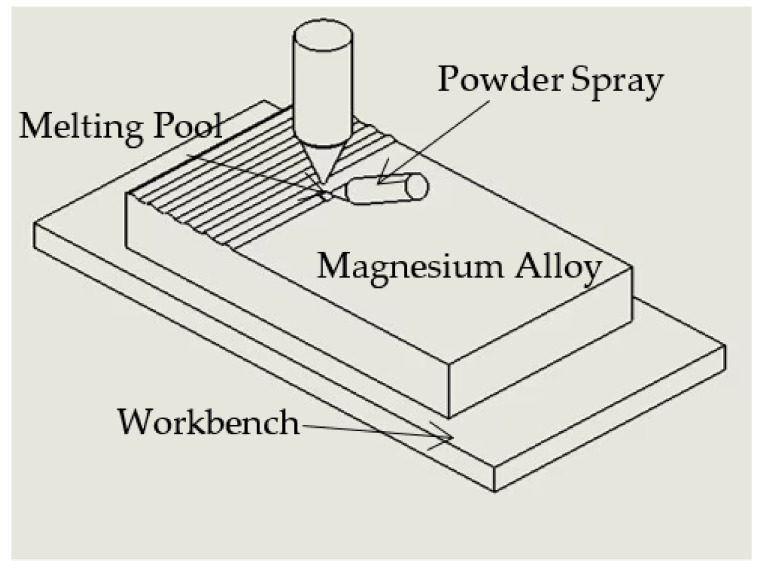
Schematic illustration of the laser cladding process.

For the cladding treatment, a YAG fiber laser having the maximum power of 6 kW was employed. Laser cladding used synchronous powder feeding. Studies have shown that high output power is conducive to the formation of a more uniform microstructure, but too high power or too low scanning speed will lead to surface evaporation and pit formation, while too low power or too high scanning speed will lead to insufficient melting, inhomogeneous particle distribution and failure of the bonding interface. Following several experiments, the following technical settings were determined to be optimal: 1 kW laser power, 6 mm/s scanning velocity, 3 mm laser beam size, and 1.2 r/min powder feeding rate. Meanwhile, high-purity argon was employed as the shielding gas due to the easy oxidation of the laser cladding procedure. Figure 1 shows a schematic illustration of the laser cladding process.

### 2.3. Microstructure and Phase Analysis

The specimens were machined into size of 10 × 10 × 5 mm^3^ by wire cutting for testing. From the cross-section of the laser-clad samples, microstructural investigations were performed using a GeminiSEM 300 scanning electron microscope (SEM). Phase constituents were analyzed using an Ultima IV multipurpose X-ray diffractometer (XRD), with the scanning 2θ in a range from 10 to 90°. Surface morphologies of the as-cladded coatings, as well as the coatings after wear test and corrosion test, were characterized by optical microscope (OM) and SEM, respectively.

### 2.4. Investigation of the Mechanical, Wear and Corrosion Properties

A TMVS-1 Vickers microhardness tester was used to measure the microhardness, with a force of 100 gf (0.98 N) and hold for 10 s during measurement. Each microhardness point was determined by performing five consecutive measurements on the coated cross section at the same depth and averaging the results. The sliding tribological test was performed in dry condition using an MPX-3G pin-disk friction wear tester. The grinding process lasted 15 min with a 50 N experimental load. The wear volume and worn surface morphologies have been analyzed following the previous studies [29,30]. Use epoxy resin to encapsulate the sample to be tested, and use CHI660e electrochemical workstation to test the Tafel polarization curve. The working electrode was the sample, the auxiliary electrode was a platinum sheet, and the reference electrode was a saturated calomel electrode. The electrolyte employed was a 3.5% NaCl solution.

## 3. Results and Discussion

### 3.1. Microstructural Study

**Figure 2:**
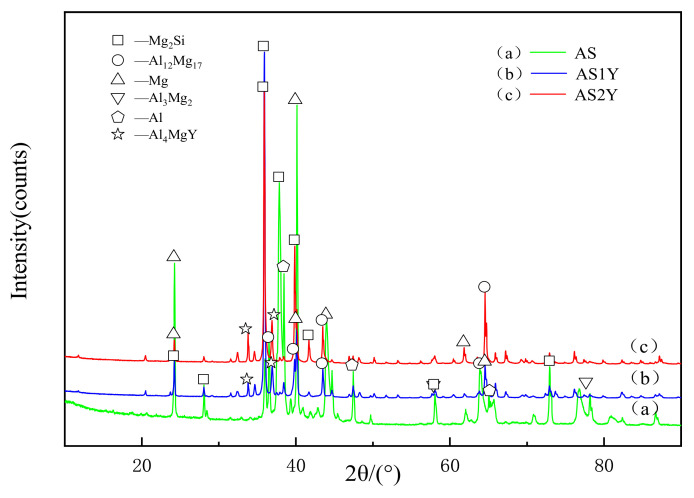
XRD patterns of the laser cladded coatings on AZ91D substrate: (**a**), (**b**) and (**c**).

The XRD patterns of the laser-clad Al-12Si coating and the Y_2_O_3_-modified coatings are shown in Figure 2. It can be seen that α-Mg and Mg_2_Si phases have formed in the unmodified coating, and the diffraction peak intensities of these two phases are relatively high. Moreover, they are also accompanied by the production of a certain amount of Al_12_Mg_17_ and Al_3_Mg_2_ phases. In addition to Mg_2_Si and Al_12_Mg_17_ phases, Al_4_MgY is newly formed phase in the Y_2_O_3_ modified coating. Furthermore, no other contamination phases were observed within the coatings, which might be because rare earth elements have a purifying impact on the molten pool during process.

**Figure 3:**
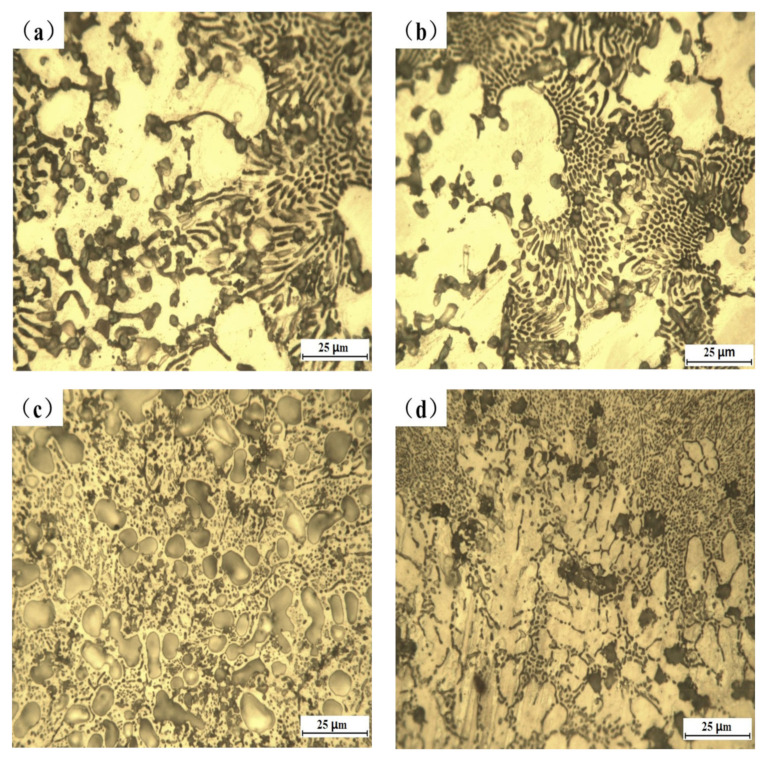
Optical micromorphology of cladded samples: (**a**) AS, (**b**) AS1Y, (**c**) AS1.5Y, and (**d**) AS2Y.

Figure 3 displays the optical micrographs of the Al-Si powder coated samples with and without Y_2_O_3_. By comparing the morphologies of these four samples, it can be clearly observed that the eutectic structure and particle phase of the coating without adding rare earth oxides are coarse and massive, and the distribution of the microstructure is relatively concentrated. After adding Y_2_O_3_, the thick eutectic structure and massive phase structure become small and distributed uniformly, and the dendritic structure is significantly refined. The results show that rare earth has an obvious effect on the microstructure refinement of the laser cladded layer.

**Figure 4:**
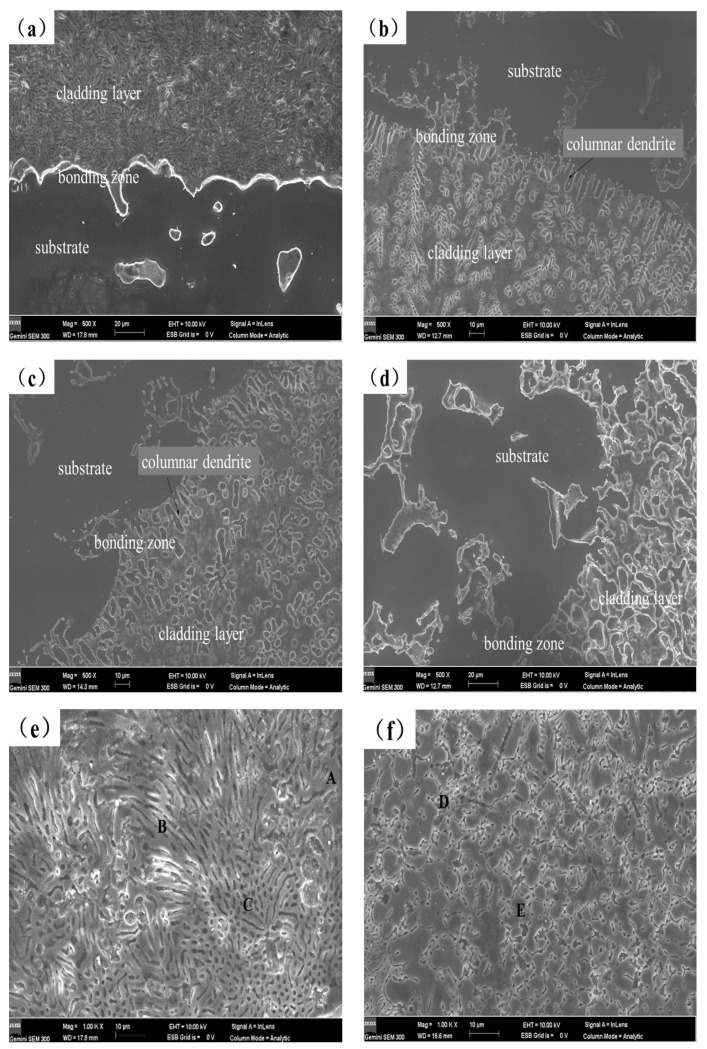
SEM microstructures of laser cladded coatings in this study: (**a**) AS, (**b**) AS1Y, (**c**) AS1.5Y, (**d**) AS2Y, (**e**) AS under magnification (Positions A, B and C are the EDS analysis area), and (**f**) AS2Y under magnification (Positions D and E are the EDS analysis area).

Figure 4 displays the cross-sectional morphology of the coating as seen by scanning electron microscopy. From the figure, it can be seen that the coating is divided into the cladding layer, the bonding zone and substrate. The results show that there is a strong metallurgical connection at the contact between the molten cladding layer and the substrate. There are no noticeable pores or flaws in any of the cladding layers. From each cladding layer area, the dendrite structure obviously shows a tendency to grow towards the top of the cladding layer. The cladding layer has a large number of dendrites and part of the cellular structure as shown in Figure 4a. Figure 4b,c show that there are clear columnar dendrites close to the substrate at the bottom of the cladding layer, which should be due to the large heat dissipation rate at the substrate. The temperature gradient becomes smaller, and the nucleus-forming rate is improved, thus resulting in the grain growth in a very short time with relatively strong directionality [31]. Furthermore, the cladding layer microstructure changes from coarse directed dendrites to fine equiaxed dendrites. The cladding layer structures are changed due to the addition of Y_2_O_3_, which acts as the nucleation center of dendrites [8], preventing dendrite coarsening and decreasing the dendrite arm spacing underneath the molten pool’s non-equilibrium solidification and the influence of convective disturbance. As shown in Figure 4b, some dendritic and rod-like structures are distributed within the cladding coating layer, and the dendrite distribution is aggregated. Figure 4c shows that the rod-like structure in the cladding layer becomes short and thick with a little petal-like structure, and the structure distribution is more uniform. Figure 4d displays the AS2Y cladding layer. The directionality of the dendritic structure in the coating is no longer obvious from Figure 4d, and the eutectic structure tends to be more refined. The microstructure of the Al-Si + Y_2_O_3_ coating exhibits a dendritic eutectic structure with varying degrees of refinement in comparison to the coating without Y_2_O_3_, and the dendritic eutectic tends to distribute uniformly, which would be beneficial to a high microhardness, low wear rate and great corrosion performance of the coatings [26].

As shown in Figure 4e, three typical microstructural areas of A, B, and C are selected on the surface of the AS coating for SEM-EDS chemical composition analysis. Among them, zones A and B are blocky and strip-shaped with lighter color, respectively. The microstructures measured in area C are small particles with darker color. Based on the test results in Table 3, it is found that the structure measured in the strip region B is rich in Mg and Al elements, with an atomic ratio of nearly 1.4:1, so it can be inferred that the intermediate phase in the region is mainly Al_12_Mg_17_. It can be seen from the measurement results of C that the main elements in this area are Mg and Si. Combined with XRD analysis, it can be concluded that the substances in this area are mainly Mg_2_Si. The high magnification SEM image of AS2Y coating presents that the microstructure morphologies are fine stripes, and the structures are more dense and uniform, which have been shown in Figure 4f. Combined with the SEM-EDS measurement results in Table 3, it can be seen that most of the Y_2_O_3_ particles have dissolved and decomposed into Y due to the strong metallurgical reaction. The Y atoms in the cladding coating are primarily distributed at the grain boundaries due to the greater atomic radius of Y of 0.18 nm [32]. Rare earth element Y acts as a non-homogeneous nucleus to inhibit grain growth by dragging effect on grain boundaries, thus inhibiting the growth of dendritic grains [33]. In addition, the high melting point of Y_2_O_3_, because of the increased content, it requires more energy to disintegrate and reduces the molten pool lifespan, thus the crystal does not have enough time to grow [16].

### 3.2. Microhardness Analysis

**Figure 5:**
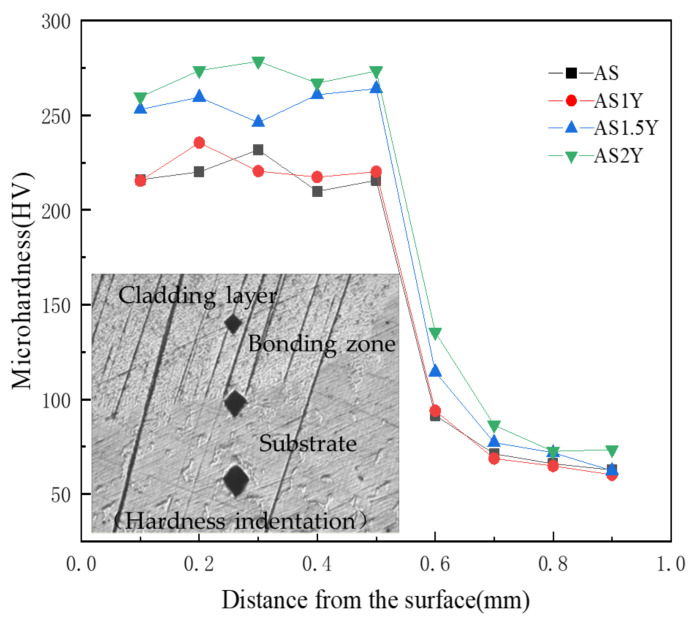
Microhardness distribution across the cross-section of coatings.

Figure 5 shows the microhardness measurement results and indentation morphologies of the laser cladded sample sections. The curves in Figure 5 show that all the cladded samples have a similar tendency in terms of the microhardness distribution over the coating depth, which is consistent with the variation of the indentation dimension. The test results show that the test indentation steadily increases from the upper surface of the cladding to the substrate, indicating that the cladding surface has the highest hardness. Measurements of the hardness of coatings containing Y_2_O_3_ in comparison to the AS cladding layer show a significant improvement. The microhardness of all cladded coatings is higher than that of the substrate, which is about 75 HV, and the average microhardness of the cladding layer of AS2Y coating reaches the highest (about 270 HV), and the average microhardness of the AS cladding layer is only 220 HV. The addition of Y_2_O_3_ can refine the grain and introduce more grain boundaries. The grain boundary reinforcement and dislocation strengthening are conducive to the improvement in the coating microhardness. Additionally, the production of intermetallic compounds Mg_2_Si and Al_4_MgY has a favorable impact on enhancing the hardness of the laser-clad layer, as evidenced by the examination of microstructure, XRD, and SEM-EDS data [4].

### 3.3. Wear Analysis

**Figure 6:**
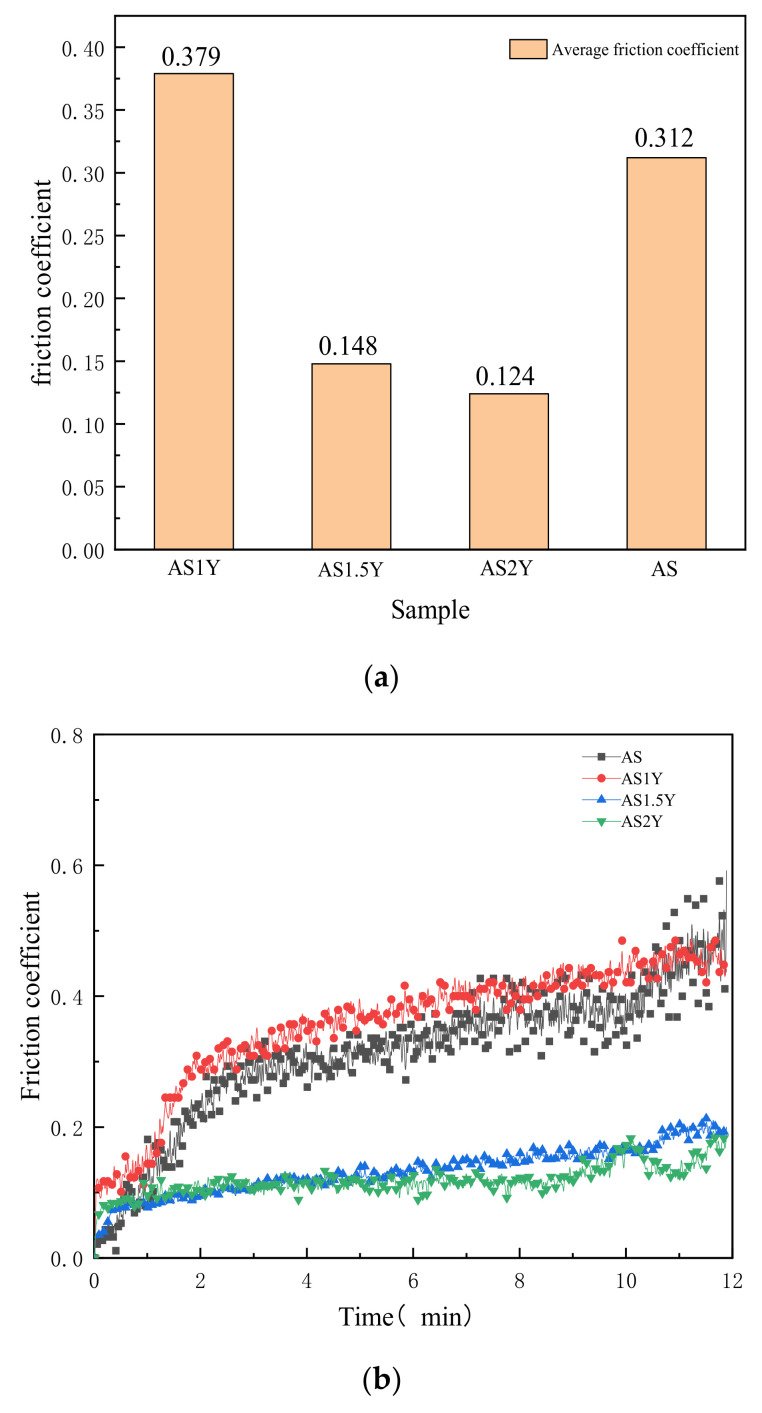
Comparison of the friction coefficient between the unmodified and Y_2_O_3_-modified coatings: (**a**) average friction coefficient, and (**b**) friction coefficient evolution history.

A key indicator of abrasion resistance is the friction coefficient. Figure 6a shows the average friction coefficient of various coatings investigated in the current study. The average friction coefficient of the coating shows a lower trend with increasing Y_2_O_3_ content, which may indicate a steady increase in the wear resistance of the coating. The transient friction coefficients versus time are shown in Figure 6b. As can be seen from the figure, the friction coefficient rises rapidly with increasing friction time, and then tends to stabilize. The rise in abrasive dust at the surface, which alters the dimension of the practical contact area and the localized contact load, may be the cause of the rapid increase in friction coefficient [34]. Because floating slag and gas can inevitably generate impurities and pores in the surface layer, AS1Y coating’s average friction coefficient is a little bit higher than coatings without Y_2_O_3_ addition. However, the overall dynamic friction coefficient variation is smoother for the AS1Y coatings compared to the coatings without Y_2_O_3_. Rare earth Y elements could present wetting effect within the microstructure on account of its surface activity and mobility in the molten pool, which makes the microstructure distribution more uniform. According to the results, the friction coefficient of AS2Y coating displays the lowest value, which is about 60% lower than that of AS coating, and the fluctuation is the most stable, showing good wear resistance. It is worth to note that the friction coefficients obtained in this study in Figure 6 are much lower than the generally reported dry sliding friction coefficient in a range of about 0.5–0.8 for most of metallic materials [35,36,37,38]. According to the results displayed in Figure 6, it has been found that addition of Y_2_O_3_ acts as a solid lubricant during dry sliding and is effective into reducing the friction coefficient. The friction coefficient of the iron-based alloy coating prepared by Wang et al. [39] was reduced by about 0.1 due to the addition of La_2_O_3_. He et al. [40] fabricated Al-TiC-CeO_2_ coatings with a minimum friction coefficient of about 0.5. The friction coefficient of the ASY coatings prepared in this work is significantly reduced.

**Figure 7:**
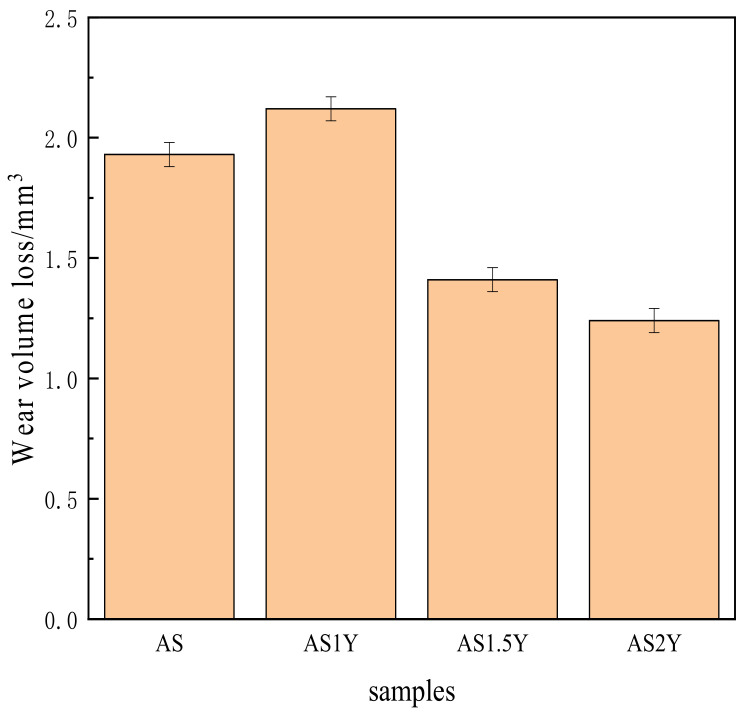
Influence of Y_2_O_3_ addition on the wear volume losses of the coatings.

Figure 7 shows the wear volume loss of the cladding layer after 15 min of anti-grinding. The figure clearly shows that the AS2Y cladding layer’s wear volume loss is substantially smaller than that of the AS coating, demonstrating that the addition of Y_2_O_3_ can somewhat reduce the coating’s wear loss and increase its wear resistance. In fact, the evolution tendency of the wear volume loss in Figure 7 is consistent with the microhardness results in Figure 5. According to Deng et al. [29,30,38], a higher wear resistance or lower wear volume loss is generally obtained by increasing the mechanical strength and microhardness of a metallic material.

**Figure 8:**
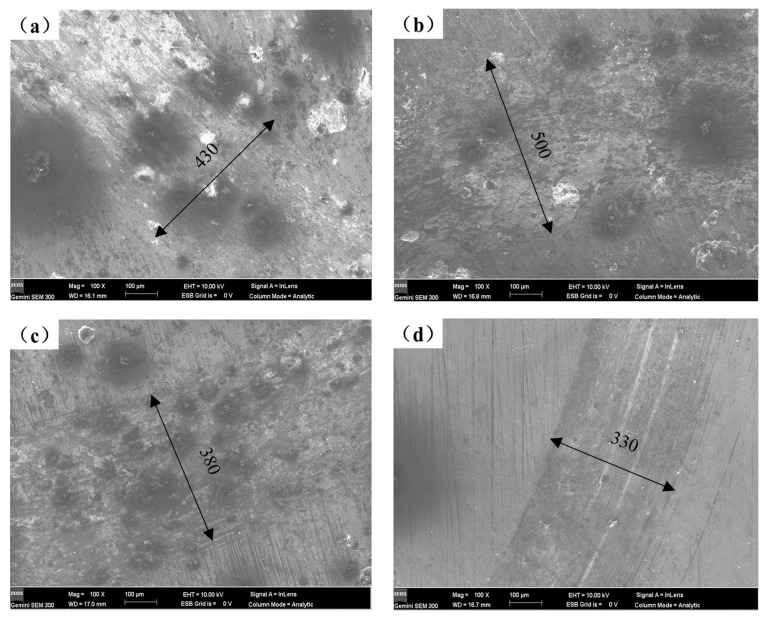
Worn surface morphologies of different coatings: (**a**) AS, (**b**) AS1Y, (**c**) AS1.5Y, and (**d**) AS2Y.

The worn surface morphologies of various coatings as seen under SEM are displayed in Figure 8. Comparing the wear scars of the four samples, it is observed that under the same magnification, the width of the wear scars gradually decreases with the increase of Y_2_O_3_ content. The wear scar width of the coating falls from approximately 500 to 330 μm, as illustrated in Figure 8b–d, as the Y_2_O_3_ content rises from 1 to 2 wt%. The wear mechanism of the coatings investigated is a combination of abrasive and adhesive wear [2], and the adhesive zones are clearly visible. As seen in Figure 8b–d, the degree of wear of coatings containing Y_2_O_3_ gradually declines, with minor wear and micro-cuts serving as the primary wear features. The improvement in wear resistance is due to the formation of a refined Mg2Si phase in the microstructure, which acts as a pressure-bearing agent on the friction surface. Meanwhile, the addition of Y_2_O_3_ may improve the microstructure of the coating and make the hard phase dispersed distribution, thus improving the uniformity of the microstructure. As mentioned above, the microstructure of the coating is altered by the addition of Y_2_O_3_, going from columnar dendrites to a fine-grained cellular network structure, greatly increasing the hardness of the composite coating. Therefore, it is more difficult for the friction pair to press into the coating surface, and the wear resistance of the coating can be significantly improved. In addition, Y_2_O_3_ may hasten the convection of the molten pool and encourage a uniform distribution of the hard phase, which may in part contribute to the wear resistance of the cladding layer due to the reinforcement of the dispersion.

### 3.4. Corrosion Resistance Analysis

**Figure 9:**
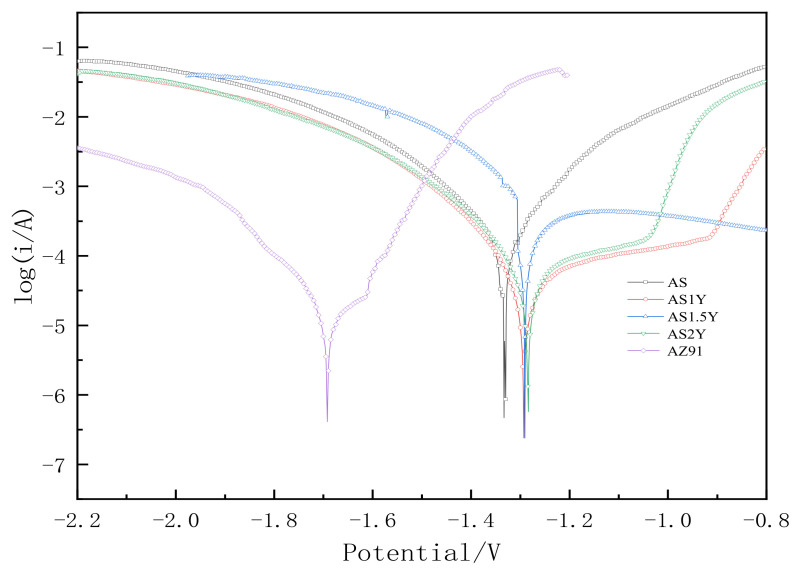
Potentiodynamic polarization curve.

**Figure 10:**
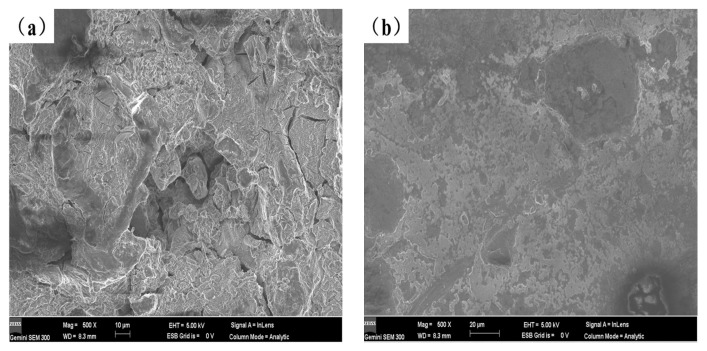

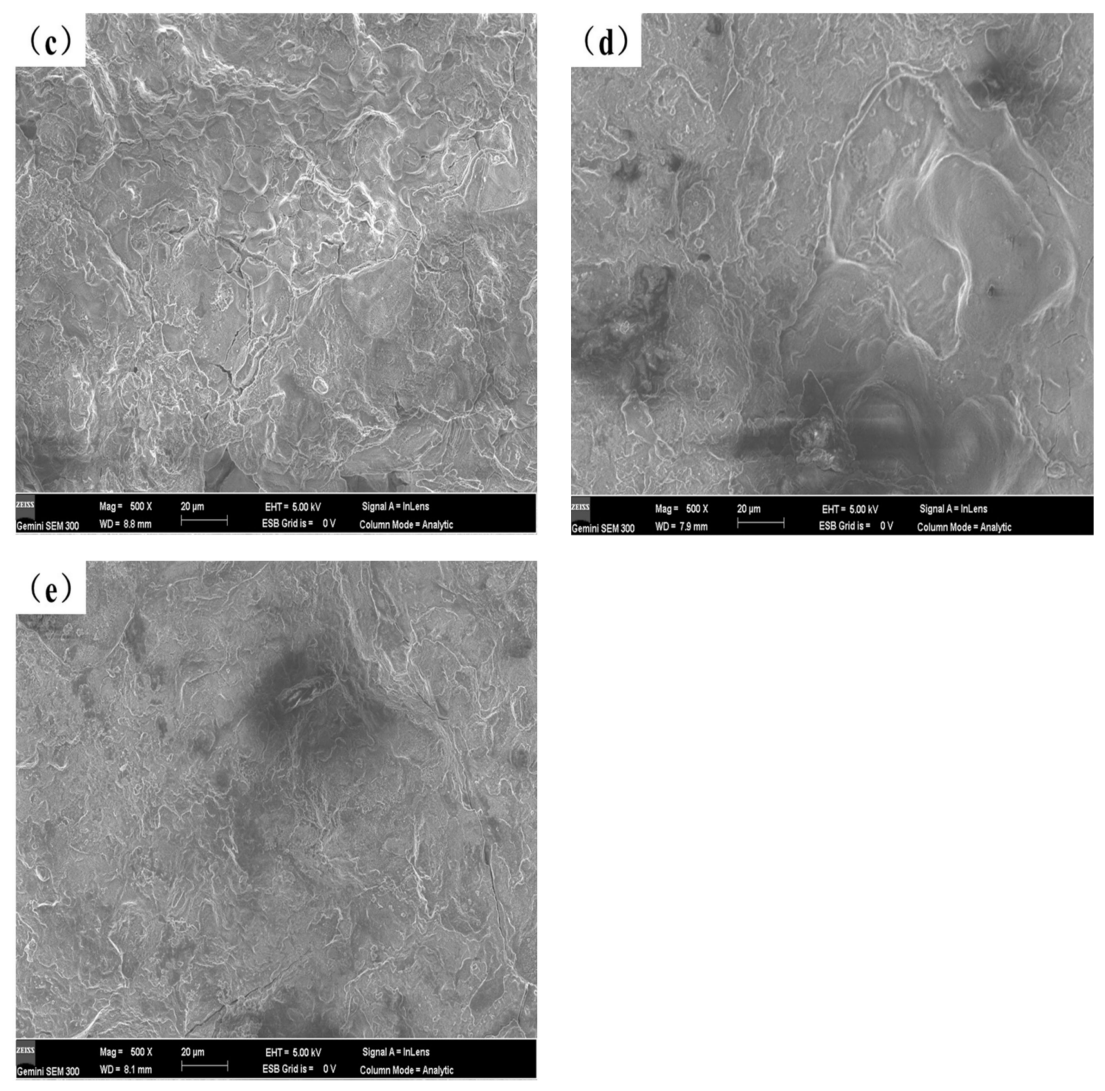
Surface morphology of different coatings after corrosion test in 3.5 wt.% NaCl solution: (**a**) AZ91D, (**b**) AS, (**c**) AS1Y, (**d**) AS1.5Y, (**e**) AS2Y.

Figure 9 depicts the potentiodynamic polarization curves of the test specimens in a 3.5 weight percent solution of NaCl at room temperature, and Table 4 lists the electrochemical parameters that were obtained from Figure 9. Lower corrosion current values correspond to lower corrosion rates and greater corrosion resistance of the coating in terms of corrosion dynamics [33]. The data in Table 4 shows that the corrosion resistance of the coating created by laser cladding on the AZ91D magnesium alloy substrate is greatly improved compared to the uncoated AZ91D, and with the content of Y_2_O_3_ increasing the corrosion current density of the coating generally increases first and then decreases. When the Y_2_O_3_ content in the coating reaches 2%, the corrosion potential of AS2Y sample is the highest (−1.284 V), and the corrosion current density reaches the lowest (7.027 × 10^−5^ A/cm^2^) among all the samples tested, which indicates the best corrosion resistance. Figure 10 shows the surface corrosion morphologies of AZ91D, AS and AS2Y samples after salt-immersion tests in 3.5 wt.% NaCl solution for 72 h. It can be observed that the corrosion surface of AZ91D in Figure 10a tends to develop vertically accompanied by deep and large corroded pores, which indicates the poor corrosion resistance. In the case of AS sample in Figure 10b, the occurrence of homogeneously uniform features with few pitting corrosion sites is evidenced at the specimen surface. Figure 10c shows no obvious pitting, with some micro-cracking on the surface. Figure 10d corrosion surface smooth without cracks produced, a very small part of the surface shows micro-pitting. In contrast, the surface of AS2Y sample in Figure 10e presents quite flat characteristics without pitting corrosion sites, indicating a superior corrosion resistance. Thus, adding Y_2_O_3_ to coatings is a viable technique to improve the corrosion behavior of AZ91D magnesium alloy, much like adding calcium to AZ31 magnesium alloy [41].

The results show that the improved corrosion resistance of the Y_2_O_3_-modified coatings is mostly the result of structural changes within the coatings, suggesting that the corrosion of the Y_2_O_3_-modified specimens is in the passivation phase [26]. Shao et al. [42] recently reported the link between the microstructure and corrosion behavior of the magnesium alloy AZ91D. The introduction of rare earth elements into coatings has the effect of purifying the melt, purifying grain boundaries and inhibiting the growth of crystal columns for the molten pool metal in the laser cladding process [43], which has also been observed in the cross-sectional morphologies of the coatings mentioned above. As the Y_2_O_3_ content increases, the content of non-metallic inclusions and impurities in the coating structure are significantly reduced, and the metallurgical structure of the coating tends to denser, especially the existence of porosity and inhomogeneity would be reduced. Similar phenomenon has also been found by Qi et al. [44]. Meanwhile, the coarse dendrites, reticulated eutectic structures and bulk grains in the Al-Si + Y_2_O_3_ cladding layer tend to be refined. Thus, in general, refined grain size conduces to suppression of corrosion rate. In addition, the dispersion distribution of compound phases such as Mg_2_Si and Al_4_MgY plays a positive influence in improving the average corrosion potential and delaying the galvanic corrosion of the cladding layer, and the formation of these phases greatly reduces the amount of α-Mg used as the anode, which reduces the occurrence of intergranular corrosion, thereby lowering the rate of corrosion and increasing the corrosion resistance of samples.

## 4. Conclusions

By using laser cladding technology, Al-Si + Y_2_O_3_ coatings were created on the surface of AZ91D magnesium alloys, and the impact of Y_2_O_3_ content on the microstructure and characteristics of the Al-Si coatings was investigated. The outcomes of the trial revealed that:

(1) The microstructures of Al-Si + Y_2_O_3_ cladding layer include α-Mg, Mg_2_Si, Al_4_MgY phases and a small amount of Al_12_Mg_17_ phase. With the increase of the amount of Y_2_O_3_ added, the coarse dendrites, reticulated eutectic structures and massive phases in the Al-Si + Y_2_O_3_ cladding layer tend to be refined and gradually uniform distribution.
(2) With an increase in Y_2_O_3_ content, the microhardness of the Al-Si coating increases. When the content of Y_2_O_3_ reaches about 2 wt.%, the microhardness of the coating reaches the greatest with an value of about 270 HV.
(3) Friction experiments show that increasing the Y_2_O_3_ content, the average friction coefficient and the wear scar width of the coating have decreased. In addition, the Y_2_O_3_ addition promotes the reduction of wear volume loss of the cladding layer, which effectively decreases the wear rate of the magnesium substrate.
(4) The Y_2_O_3_-modified coatings have greater corrosion resistance than the untreated coatings. The addition of Y_2_O_3_ promotes the reduction of localized corrosion sites and makes the corrosion surface smoother, implying that the corrosion resistance increases significantly.

## Figures and Tables

**Table 1:** Chemical composition (wt.%) of AZ91 magnesium alloy.

Al	Zn	Mn	Si	Fe	Cu	Ni	Be	Mg
9.3	0.63	0.32	0.05	0.003	0.021	0.001	0.001	Bal.

**Table 2:** Samples and composition (wt.%) of coatings.

Title 1	Title 2
AS	Al-12Si
AS1Y	Al-12Si + 1Y_2_O_3_
AS1.5Y	Al-12Si + 1.5Y_2_O_3_
AS2Y	Al-12Si + 2Y_2_O_3_

**Table 3:** Chemical compositions of the microstructure analyzed by SEM-EDS (wt.%).

Position	Al	Mg	Si	Y	O
A	41.0	58.1	0.9	0	0
B	42.1	51.3	6.6	0	0
C	14.7	45.8	39.6	0	0
D	74.7	3.6	19.6	2.0	0.1
E	65.6	4.6	28.7	1.2	0

**Table 4:** Electrochemical parameters calculated from the results in Figure 9.

Sample	$E_{corr}/V$	$I_{corr}/\left(A\cdot cm^{-2}\right)$	$\beta_{a}/V$	$\beta_{c}/V$	$R_{p}/\Omega$
AZ91D	−1.692	1.22 × 10^−3^	0.376	−0.164	40.696
AS	−1.333	7.466 × 10^−5^	0.127	−0.143	391.706
AS1Y	−1.293	9.85 × 10^−5^	0.330	−0.122	393.162
AS1.5Y	−1.287	8.737 × 10^−5^	0.296	−0.151	497.988
AS2Y	−1.284	7.027 × 10^−5^	0.391	−0.129	600.158

## Data Availability

Not applicable.
